# Test–Retest Reliability of Physiological Variables During Submaximal Seated Upper-Body Poling in Able-Bodied Participants

**DOI:** 10.3389/fphys.2021.749356

**Published:** 2021-11-30

**Authors:** Marlou Ettema, Berit Brurok, Julia Kathrin Baumgart

**Affiliations:** ^1^Centre for Elite Sports Research, Department of Neuromedicine and Movement science, Norwegian University of Science and Technology, Trondheim, Norway; ^2^Department of Human Movement Sciences, Faculty of Behavioural and Movement Sciences, Vrije Universiteit Amsterdam, Amsterdam Movement Sciences, Amsterdam, Netherlands; ^3^Department of Physical Medicine and Rehabilitation, St. Olav’s University Hospital, Trondheim, Norway

**Keywords:** submaximal exercise test, upper-body exercise, aerobic, endurance, consistency

## Abstract

**Purpose**: To investigate the test–retest reliability of physiological variables across four different test days and four different submaximal exercise intensities during seated upper-body poling (UBP).

**Methods**: Thirteen abled-bodied, upper-body trained men (age 29±3years; body mass 84±12kg; height 183±5cm) performed four submaximal 4-min stages of seated UBP on four separate test days. The four submaximal stages were set at individual power outputs corresponding to a rating of perceived exertion of 9, 11, 13, and 15. The absolute reliability for pairwise test-day comparisons of the physiological variables was investigated with the smallest detectable change percentage (%SDC) and the relative reliability with the interclass correlation coefficient (ICC).

**Results**: Absolute and relative reliability across test-day comparisons and submaximal stages were moderate to excellent for all variables investigated (V̇O_2_ – %SDC range: 5–13%, ICC range: 0.93–0.99; HR – %SDC range: 6–9%, ICC range: 0.91–0.97) other than blood lactate, for which absolute reliability was poor and relative reliability highly variable (%SDC range: 26–69%, ICC range: 0.44–0.92). Furthermore, absolute and relative reliability were consistent across the low-to-moderate exercise intensity spectrum and across test days.

**Conclusion**: Absolute and relative test–retest reliability were acceptable for all investigated physiological variables but blood lactate. The consistent test–retest reliability across the exercise intensity spectrum and across test days indicates that a familiarization period to the specific exercise modality may not be necessary. For generalizability, these findings need to be confirmed in athletes with a disability by future large-scale studies.

## Introduction

Aerobic endurance performance is determined by maximal oxygen uptake (V̇O_2max_), oxygen uptake (V̇O_2_) at the anaerobic threshold, and exercise efficiency (Bosquet et al., 2002; Bentley et al., 2007; Joyner and Coyle, 2008; Poole et al., 2016). While V̇O_2max_ is established through maximal tests to exhaustion, submaximal testing provides information on the anaerobic threshold and exercise efficiency. For able-bodied athletes, the most common exercise modality for testing aerobic endurance performance is a treadmill or cycle ergometer. For endurance athletes with impairments of the lower extremities and/or trunk, upper-body exercise modalities, such as arm crank or wheelchair ergometers, are frequently used (Grange et al., 2002; Bougenot et al., 2003; Price et al., 2011; Schrieks et al., 2011; Gauthier et al., 2017b). In a sports context, testing should be performed in specific exercise modalities to ensure that the investigated variables are reflective of the demands of the sport in question (Pechar et al., 1974; Roels et al., 2005). This also applies to sitting Para cross-country skiers, Para biathletes, and Para ice hockey players, for whom the seated upper-body poling (UBP) modality is sport-specific (Baumgart et al., 2017).

During submaximal testing at a given power output (PO) or speed, a lower oxygen uptake (V̇O_2_), lower heart rate (HR), and reduced blood lactate concentration (BLa) may indicate an improvement in training status. In addition, a lower metabolic rate at a given submaximal PO indicates increased exercise efficiency. However, the test–retest reliability of these variables needs to be established before changes between exercise test instances can be attributed to true training effects – and not just to artifacts of natural variability. To date, good-to-excellent test–retest reliability of physiological variables has been established during submaximal (Van’t Hul et al., 2003; Eng et al., 2004) and maximal lower-body exercise (McArdle et al., 1972; Beekley et al., 2004; Sartor et al., 2013; Ekblom-Bak et al., 2014). Furthermore, during maximal upper-body exercise testing, the reliability of peak physiological variables was shown to be good to excellent (Leicht et al., 2009, 2013; Flueck et al., 2015; Baumgart et al., 2017; Gauthier et al., 2017a). The only two studies that investigated the test–retest reliability of physiological variables during submaximal upper-body exercise, did so at moderate intensity, in both patients with spinal cord injury and able-bodied participants, and employed an arm crank ergometer (Hol et al., 2007; Bulthuis et al., 2010). While this has not yet been investigated during upper-body exercise, the variability of physiological responses at low intensity compared to moderate/high intensity may be larger due to decreased efficiency. In addition, it is commonly advised to perform a familiarization session to reduce learning effects before continuing with the actual testing (Dean et al., 1989;Atkinson and Nevill, 1998; Hopkins, 2000). However, the extent of the differences in physiological responses between the first and consecutive test days, and whether a familiarization session could potentially be dropped, has not yet been investigated. This is important knowledge especially for elite athletes, who have limited time to engage in these familiarization sessions.

Para sitting sport athletes with different disabilities are a relatively small group with a larger heterogeneity in physiological responses compared to abled-bodied athletes. Therefore, to circumvent the challenges related to heteroscedasticity and inflated high relative reliability (Atkinson and Nevill, 1998; Hopkins, 2000), testing able-bodied upper-body trained participants to establish baselines values may be a good alternative.

The aim of this study was to investigate the test–retest reliability of physiological variables during seated UBP in able-bodied upper-body trained participants. This was done across different submaximal exercise intensities on four separate test days.

## Materials and Methods

### Overall Design

The testing consisted of four separate test days, with a minimum of 48h between each test day. All participants completed the total testing period within two weeks. Four submaximal stages were performed on each test day in the same order. The intensity of the four submaximal stages was determined for each individual participant by establishing the PO that corresponded with a RPE of 9, 11, 13, and 15 on the 6–20 Borg scale (Borg, 1982). RPE was initially used during T1 to ensure that participants covered a similar range of perceived exercise intensities (Tucker, 2009). The corresponding PO was determined during the first test day for each individual and kept constant during the remaining three test days. To minimize the effect of diurnal fluctuations, participants performed all tests at approximately the same time of day (Atkinson and Reilly, 1996; Reilly et al., 2007).

### Participants

Thirteen able-bodied, upper-body trained men participated in this study (Table 1). All participants were recreationally active cross-country skiers or biathletes, who trained approximately 2–3 times per week employing exercise modalities involving upper-body musculature, such as the double poling technique. Note that the data were obtained as part of a previously published study by Brurok et al. (2019), who investigated the influence of different incremental tests on peak physiological responses. The participants were asked not to consume any large meals 2h before testing, to refrain from caffeine consumption on the day of testing and not to undertake strenuous exercise 24h prior to testing. All participants signed an informed consent prior to participating in the study. Ethical approval was obtained from the Norwegian Centre for Research Data (ID 51228). All participants were informed about the possibility to withdraw from the study at any point in time without specific reasons, and the data collection was performed in accordance with the declaration of Helsinki (World Medical Association, 2013).

**Table 1:** Characteristics of the 12 male, able-bodied upper-body trained participants.

Participant	Age (years)	Body height (cm)	Body mass (kg)
1	31	176	75.5
2	29	187	96.0
3	26	185	67.4
4	29	190	97.0
5	26	186	77.1
6	26	187	103.0
7	27	182	95.1
8	34	187	83.6
9	29	173	73.5
10	28	178	92.8
11	26	185	84.1
12	25	180	70.9
13^*^	29	183	83.7
Mean±SD	29 ± 3	183 ± 5	83.7 ± 11.9

* *The data of this participant were excluded from further analyses due to inconsistencies in power output across test days at a given submaximal stage*.

### Test Set-Up

**Figure 1:**
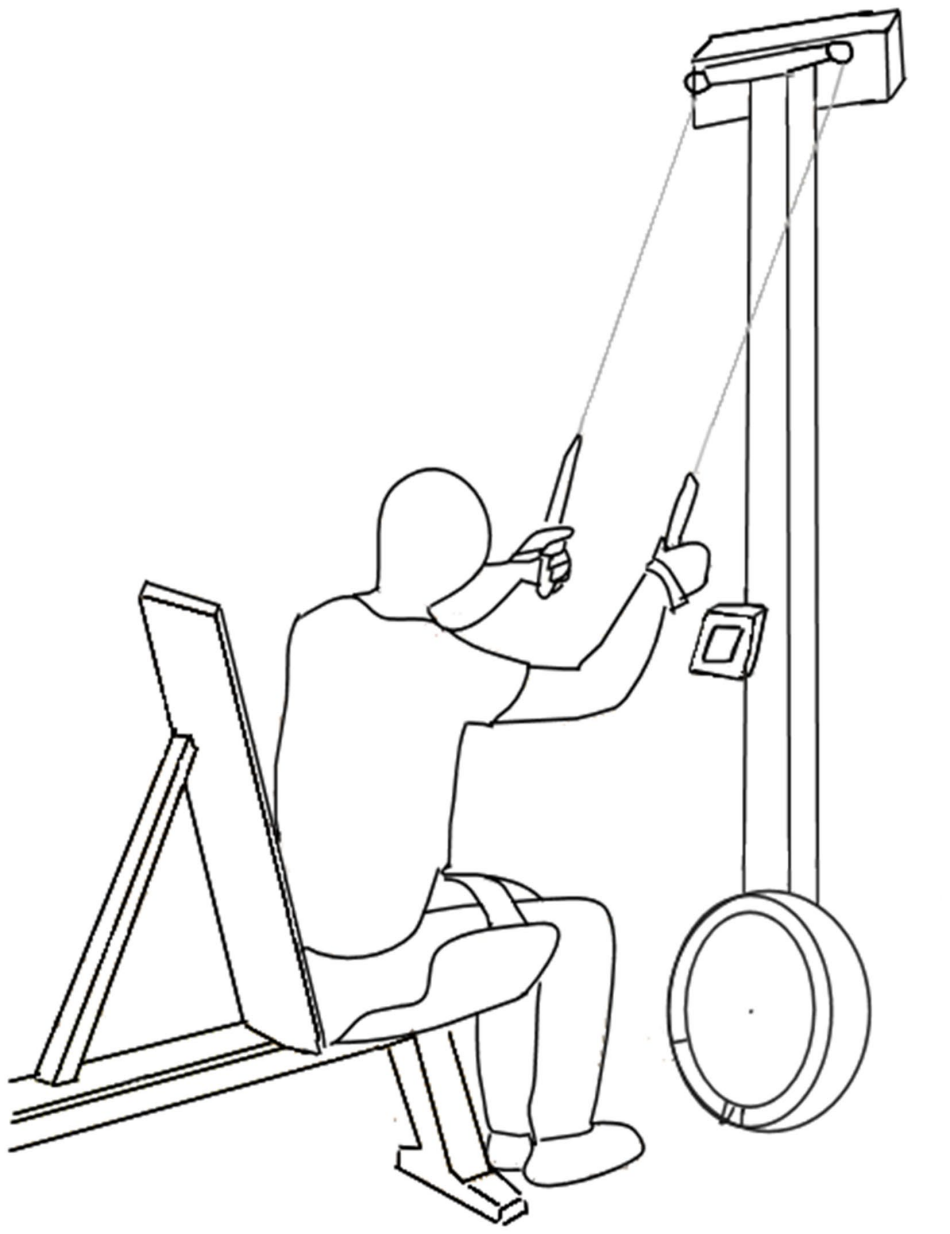
Test set-up of the Concept2 ski ergometer including the modified weightlifting bench. Permission of re-use has been granted by Baumgart et al. (2017).

The test set-up is described in more detail in Brurok et al. (2019). In brief, prior to testing, all participants were equipped with a nose clip and a mouthpiece (Hans Rudolph Inc., Kansas City, MO, United States), as well as a heart rate monitor (M400 Polar Electro Inc., Port Washington, NY, United States). The participants sat on a modified weightlifting bench in front of the Concept2 ski ergometer (Concept2, Inc., Morrisville, United States) (Figure 1), tightly strapped around the hips and thighs to secure a stable position.

### Test Protocol and Measurements

Participants performed four 4-min submaximal stages with a PO corresponding to a RPE of 9, 11, 13, and 15. The average PO at the respective RPE levels was determined individually during the first test day (T1) and then kept constant for the remaining three test days (T2, T3, and T4). Instructions on the use of the BORG scale (6–20) (Borg, 1982) for regulating RPE were provided prior to the first test session. Participants self-regulated the PO on the Concept2 ski ergometer, with the ergometer’s software (Erg-Stick Ltd., United Kingdom) continuously recording PO and stroke rate. The participants were verbally encouraged to keep the PO steady throughout the submaximal stages.

V̇O_2_, V̇CO_2_, and V̇E were determined using the Jaeger ergospirometer (Oxycon Pro, Jaeger, Viasys BV, Bilthoven, Netherlands) with an open circuit mixing chamber. The ergospirometer was calibrated against a known mixture of gases (5% CO_2_ and 15% O_2_) and against ambient air prior to each test. The calibration of the flow volume transducer was completed automatically according to the manufacturer’s instructions. The Jaeger ergospirometer was shown to be valid for recording respiratory responses during submaximal and maximal exercise (Rietjens et al., 2001). HR was continuously recorded during the tests. Directly after each submaximal stage, a 20 μL blood sample was collected from the fingertip, which was used for the analysis of BLa by a Biosen C-Line Sport lactate measurement system (EKF-diagnostic GmbH, Magdeburg, Germany).

### Data Processing

The average values of the respiratory data were recorded in 10-s intervals by the internal software of the ergospirometer. The steady state of the physiological variables (V̇O_2_, V̇CO_2_, V̇E, and HR) was calculated as the average over the last minute of the 4-min submaximal stages in Matlab version 2018a (MathWorks Inc., Natick, United States).

### Statistical Analysis

All statistical analyses were performed with the software R (R Core Team, 2013). To obtain a complete data set, missing values for the variables V̇CO_2_ (3 data points), V̇E (2 data points), HR (10 data points), and BLa (2 data points) were replaced using multiple imputations with the R *MICE* package. Two-way repeated measures ANOVA were performed to investigate differences between the four test days and four different intensities with the R *lme4* package and *lmer* function. *Post hoc* tests with Holm’s method correction were used for pairwise comparisons between the four test days for each of the four submaximal intensities employing the R *lme4* package and *lsmeans* function. Prior to performing the ANOVA, the assumption of normally distributed unstandardized residuals was assessed with histograms and Shapiro–Wilk tests. These residuals were non-normally distributed for all investigated variables (*p*<0.001). Accordingly, we log-transformed the data; however, this transformation did not impact on the results of the ANOVA, and we, hence, present the results based on the non-transformed data. An alpha level of 0.05 was used to indicate statistical significance.

### Absolute Reliability

Bland–Altman plots were used to visualize mean differences for all physiological variables across submaximal intensities between tests days (i.e., T1-T2, T2-T3, and T3-T4)±95% limits of agreement (LoA). The smallest detectable change (SDC) was used to indicate the smallest difference between test days that needs to be present to accept that it is a “true” effect and not just an artifact of natural variability or measurement error. The standard error of measurement (SEM), also known as the within-subject standard deviation, was calculated as follows: SEM=SD_diff_ . ${\left(\sqrt{2}\right)}^{-1}$ (Hopkins, 2000; Weir, 2005). The SDC was calculated with a 80% probability (i.e., SDC=SEM . 1.28 . $\sqrt{2}$ ) (Bland and Altman, 1986)_._ The percentage SDC (%SDC) was calculated for each submaximal intensity by dividing the SDC by the mean of the values obtained on the corresponding two test days (i.e., %SDC=SDC·(mean)^−1^·100). %SDC values were categorized as poor (>20%), moderate (10.1–20%), good (5.1–10%), and excellent (0–5%).

### Relative Reliability

The intraclass correlation coefficient (ICC) was calculated based on single measurement, absolute agreement, two-way mixed effects modelling (Koo and Li, 2016), employing the R *irr* package, and *icc* function. ICCs were calculated for the comparison of test days (i.e., T1-T2, T2-T3, and T3-T4) for each of the four submaximal stages. ICC values were categorized as poor (<0.5), moderate (0.5–0.75), good (0.76–0.9), and excellent (>0.9) (Weir, 2005; Koo and Li, 2016).

## Results

**Figure 2:**
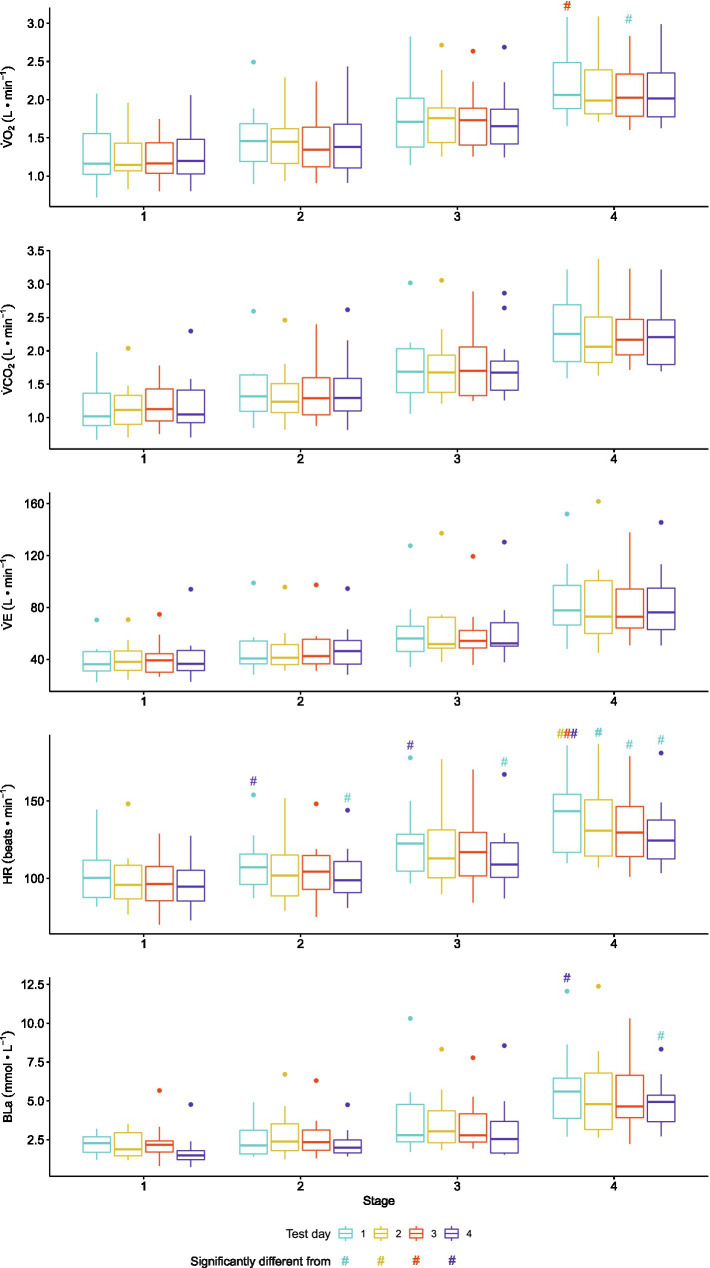
Boxplots comparing the median and interquartile range for the physiological variables collected during seated upper-body poling (UBP) on each of the four test days during each of the four submaximal intensities in 12 able-bodied upper-body trained men. Dots indicate outliers. Oxygen uptake, V̇O_2_; carbon dioxide production, V̇CO_2_; minute ventilation, V̇E; heart rate, HR; blood lactate oncentration, BLa.

All 13 participants completed the four submaximal stages on each of the four test days. However, the data from one participant were excluded because the intensities of the four submaximal stages were different between test days. The presented results are, hence, based on the data of the remaining 12 participants. Mean PO±SD was 53±22, 65±22, 85±26, and 107±24W for RPE stages 9, 11, 13, and 15, respectively. While all physiological variables significantly increased with increasing intensity (for all variables, *p*<0.001), there were no significant differences between test days at each submaximal intensity for any of the variables (*p*>0.09) but HR (*p*=0.001) (Figure 2). HR was 8–10 beats·min^−1^ lower on T4 compared to T1 for submaximal stages 2–4 (all three comparisons, *p*<0.007), with a trend towards a significant 8 beats·min^−1^ difference for stage 1 (*p*=0.09).

### Absolute Reliability

**Figure 3:**
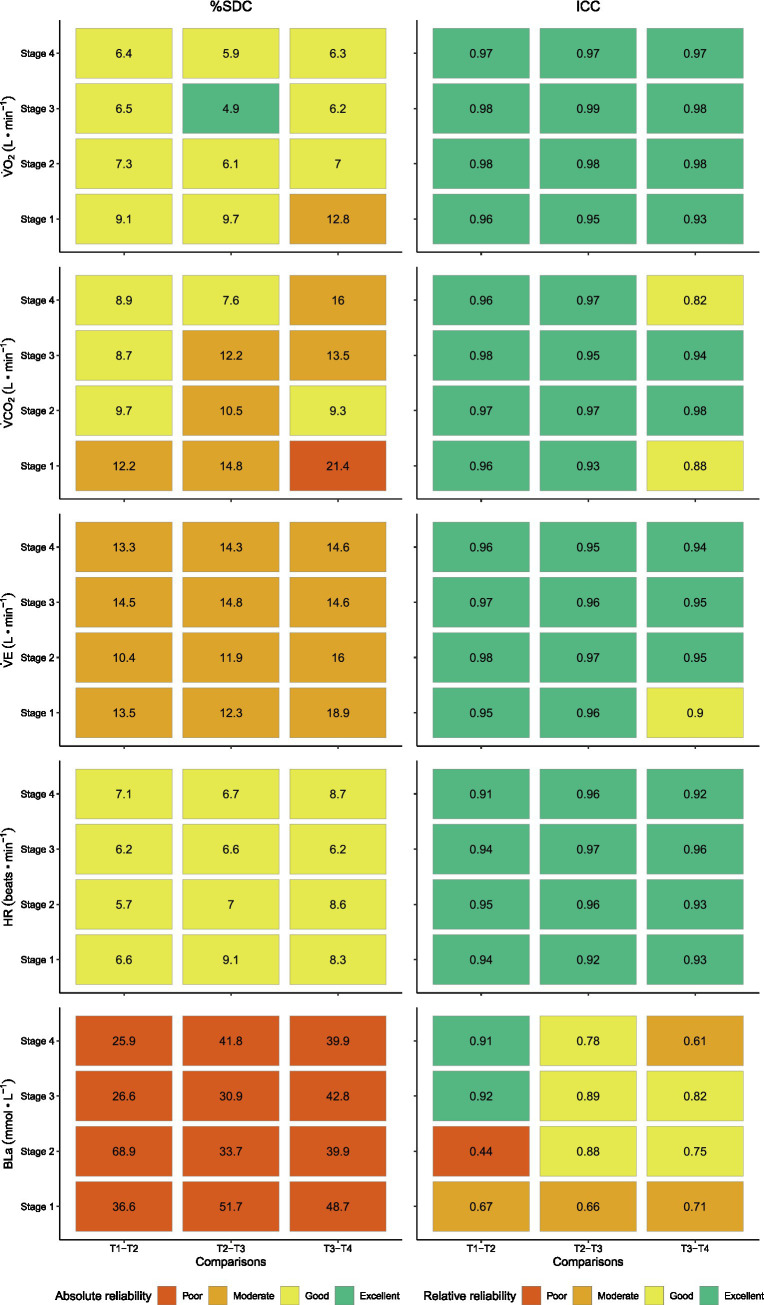
Smallest detectable change (%SDC) and interclass correlation coefficients (ICCs) for the physiological variables collected during seated UBP on each of the four test days during each of the four submaximal intensities in 12 able-bodied upper-body trained men. %SDC values were categorized as poor (>20%), moderate (10.1–20%), good (5.1–10%) and excellent (0–5%). ICC values were categorized as poor (<0.5), moderate (0.5–0.75), good (0.76–0.9), and excellent (>0.9). Oxygen uptake, V̇O_2_; carbon dioxide production, V̇CO_2_; minute ventilation, V̇E; heart rate, HR; blood lactate concentration, BLa.

**Figure 4:**
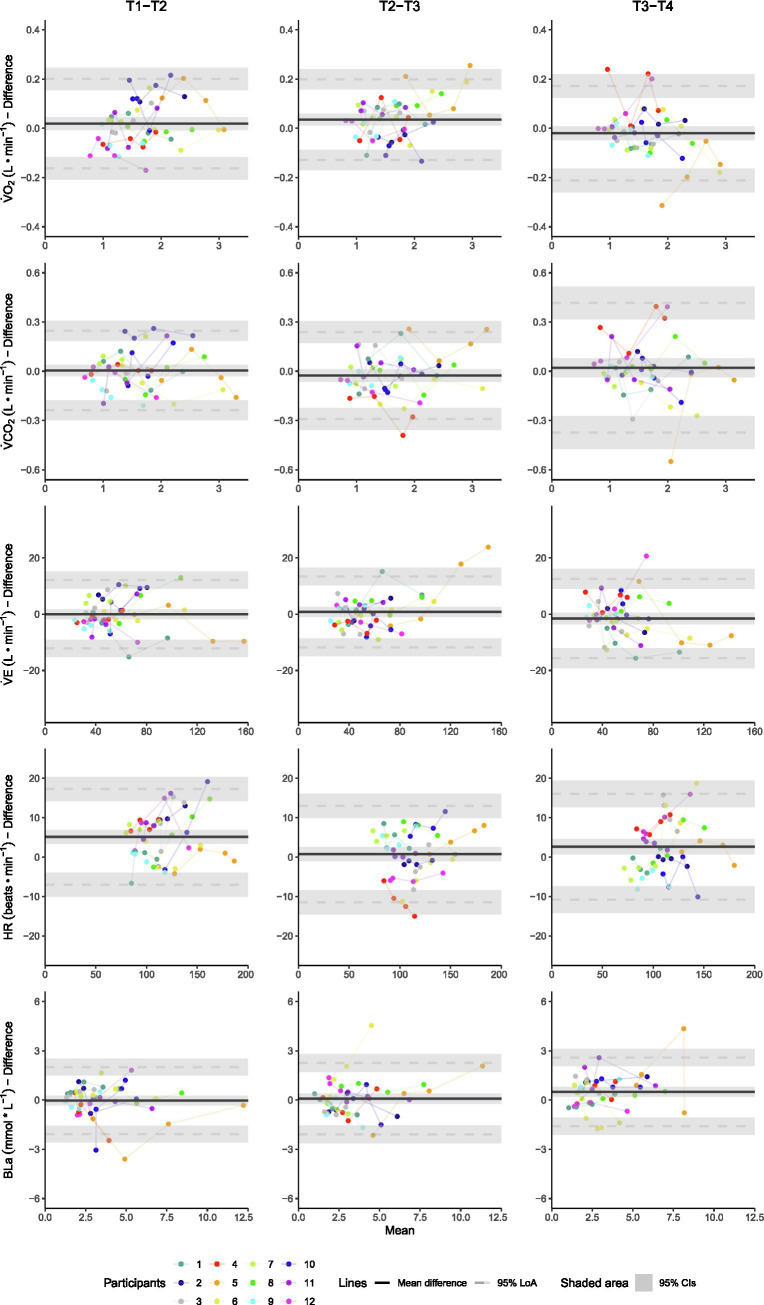
Bland–Altman plots showing the individual mean responses and differences between test days T1-T2, T2-T3, and T3-T4 for each of the physiological variables collected during seated UBP in 12 able-bodied upper-body trained men. Oxygen uptake, V̇O_2_; carbon dioxide production, V̇CO_2_; minute ventilation, V̇E; heart rate, HR; blood lactate concentration, BLa.

The %SDC indicated moderate-to-good absolute reliability for almost all physiological variables other than BLa. For BLa, poor absolute reliability was found for all comparisons between test days at each submaximal intensity with %SDCs ranging from 26 to 69% (Figure 3). Furthermore, for all investigated physiological variables, LoA and %SDC did not systematically decrease for the T2-T3 and T3-T4 compared to the T1-T2 contrasts (Figures 2, 3). In addition, for all investigated physiological variables, %SDC and LoA did not systematically decrease with an increase in UBP exercise intensity (Figures 3, 4).

### Relative Reliability

The ICC indicated good-to-excellent relative reliability for all physiological variables other than BLa. For BLa, relative reliability was generally lower and more variable ranging from poor to excellent (ICC range: 0.44–0.92) (Figure 3). As was the case for absolute reliability, also for relative reliability, the ICCs did not systematically increase for the T2-T3 and T3-T4 compared to the T1-T2 contrasts, as well as with an increase in exercise intensity (Figure 3).

## Discussion

The aim of this study was to investigate the absolute and relative test–retest reliability of physiological variables during submaximal seated UBP in able-bodied participants across different test days and exercise intensities. Absolute and relative reliabilities were moderate to excellent for all variables investigated other than BLa, for which reliability was poor. Furthermore, absolute and relative reliabilities were consistent across the low-to-moderate exercise intensity spectrum and across test days indicating that a familiarization period to UBP in specifically trained athletes may not be necessary.

Absolute reliability measures provide the possibility to investigate the degree to which repeated measurements vary for individuals. In this study, average %SDCs for V̇O_2_, V̇CO_2_, V̇E, and HR ranged from 7 to 14%, which we consider acceptable for using these variables to indicate training progress during submaximal exercise testing in the UBP modality. In contrast, the large SDCs (and variable ICCs) for BLa, which are in line with a previous study (Baumgart et al., 2020), suggest that BLa cannot be used as a reliable outcome measure during submaximal exercise testing in the UBP mode. In the context of absolute reliability, it should be noted that the %SDCs for all variables during submaximal UBP are consistently higher compared to what has been found in a similar study sample during maximal UBP (Baumgart et al., 2017), indicating that physiological responses may be more variable and less reliable at submaximal intensity.

In line with what we expected and previous studies (Hol et al., 2007; Bulthuis et al., 2010), good-to-excellent relative reliability was found for most investigated physiological variables, reflected by high ICCs. However, caution is needed in the ICC’s interpretation, as it is a ratio of the between-participant variation in relation to the within-participant variation and can be inflated merely by sample heterogeneity (Atkinson and Nevill, 1998; Hopkins, 2000). As such, the high ICCs in the current study simply indicate that the within-participant variation was relatively smaller than the between-participant variation. While we opted for a homogeneous study sample of upper-body trained individuals, physiological responses to upper-body exercise vary, especially in our case where participants exercised at different power outputs. In addition, while different POs were chosen to cover a similar perceived exertion from low-to-moderate exercise intensity, this choice may have inflated the ICCs. Concluding from the above, the interpretability of the ICC as a measure of relative test–retest reliability in upper-body testing remains limited, since even homogeneous able-bodied participants show heterogeneous responses.

Notably, reliability did not increase with an increase in submaximal intensity (i.e., indicated by a systematic decrease in the spread of the differences in the Bland–Altman plots, lower %SDCs and higher ICCs). This suggests that no additional considerations need to be made regarding exercise intensity, when the aim is to assess submaximal physiological responses across test days. Interestingly, HR was significantly lower on T4 compared to T1 across most submaximal stages, while there were no significant differences to T2 and T3. This might indicate a training effect; however, since the lower HR did not coincide with a significantly lower V̇O_2_ and BLa, we cannot conclude with certainty. Speculatively, the lower HR may be related to that the participants were more at ease in the laboratory setting during T4. Furthermore, the reliability of physiological variables was not higher for the T2-T3 and T3-T4 compared to the T1-T2 contrasts. Therefore, a familiarization session may not be needed during testing in the UBP modality if participants are, as was the case in our study, upper-body trained and accustomed to a similar movement.

### Methodological Considerations

In line with the recommendation of Hopkins (2000), we calculated the sample size needed in future studies to identify meaningful differences in absolute V̇O_2_ in a repeated measures set-up. We estimated a future sample size of 26 participants by *n*=8·SDC^2^·(SEM^2^)^−1^ for absolute V̇O_2_, and relatively similar numbers apply for most of the variables investigated in our study. This is a considerably higher number of participants than what we recruited for the current study based on *a priori* power analyses. Likely, an even higher number of participants with a disability, who display more variable physiological responses, would be needed to replicate the study and investigate the generalizability of our results. Therefore, international collaborations and multi-center approaches are needed to collect data from a sufficient number of athletes, including sub-groups of athletes with similar disabilities. Investigating sub-groups would likely be needed to circumvent inflation of the ICC and excessively large SDCs due to sample heterogeneity.

Regarding considerations for future testing of athletes with a disability, the modified weightlifting bench was constructed to allow for testing without other major modifications. Additional strapping around the upper body may be necessary for athletes with disabilities, who may lack sufficient trunk control. Furthermore, we chose RPE instead of a speed- or power-based protocol, since athletes commonly use RPE to monitor training load and as a tool to target-specific exercise intensity zones (Foster et al., 1996; Wallace et al., 2009; Paulson et al., 2015). RPE as a measure of exercise intensity is particularly useful during upper-body exercise, especially in athletes with a disability, to account for the highly variable upper-body capacities. While using a % of either peak power output or peak oxygen uptake reduces this variability to some extent, these relative values are also dependent on training status and remaining active upper-body muscle mass; thus, variability remains an issue.

## Conclusion

Absolute and relative test–retest reliability were moderate to excellent for most investigated physiological variables during submaximal UBP in able-bodied upper-body trained participants. This indicates acceptable reliability for using these variables to track training progress in a sports setting. The only exception was blood lactate, for which absolute reliability was poor and relative reliability highly variable, indicating that this variable is less suitable for tracking training progress. The consistent test–retest reliability across the exercise intensity spectrum and across test days indicates that a familiarization period to the specific exercise modality may not be necessary, at least in highly upper-body trained participants who are accustomed to a similar movement. For generalizability, these findings need to be confirmed in athletes with a disability by future large-scale studies.

## Data Availability Statement

The original contributions presented in the study are included in the article/Supplementary Material, further inquiries can be directed to the corresponding author.

## Ethics Statement

The studies involving human participants were reviewed and approved by the Norwegian Centre for Research Data (ID 51228). The participants provided their written informed consent to participate in this study.

## Author Contributions

ME, BB, and JB: conceptualization and methodology. ME and JB: formal analysis and investigation and writing – original draft preparation. BB: writing – review and editing. All authors contributed to the article and approved the submitted version.

## Funding

This study was funded by the Centre for Elite Sports Research, Department of Neuromedicine and Movement Sciences, and Norwegian University of Science and Technology. The funder had no role in the study design, how the data analyses were performed, the decision to publish or preparation of the manuscript.

## Conflict of Interest

The authors declare that the research was conducted in the absence of any commercial or financial relationships that could be construed as a potential conflict of interest.

## Publisher’s Note

All claims expressed in this article are solely those of the authors and do not necessarily represent those of their affiliated organizations, or those of the publisher, the editors and the reviewers. Any product that may be evaluated in this article, or claim that may be made by its manufacturer, is not guaranteed or endorsed by the publisher.
